# A simple method to control glycolytic flux for the design of an optimal cell factory

**DOI:** 10.1186/s13068-017-0847-4

**Published:** 2017-06-21

**Authors:** Jae Hyung Lim, Gyoo Yeol Jung

**Affiliations:** 1 Department of Chemical Engineering, Pohang University of Science and Technology, 77 Cheongam-Ro, Nam-Gu, Pohang, Gyeongbuk 37673 Korea; 2 School of Interdisciplinary Bioscience and Bioengineering, Pohang University of Science and Technology, 77 Cheongam-Ro, Nam-Gu, Pohang, Gyeongbuk 37673 Korea

**Keywords:** Glycolytic flux, *n*-Butanol, 2,3-Butanediol, Butyrate, UTR engineering, PTS

## Abstract

**Background:**

A microbial cell factory with high yield and productivity are prerequisites for an economically feasible bio-based chemical industry. However, cell factories that show a kinetic imbalance between glycolysis and product formation pathways are not optimal. Glycolysis activity is highly robust for survival in nature, but is not optimized for chemical production.

**Results:**

Here, we propose a novel approach to balance glycolytic activity with the product formation capacity by precisely controlling expression level of *ptsG* (encoded glucose transporter) through UTR engineering. For various heterologous pathways with different maximum production rates, e.g., *n*-butanol, butyrate, and 2,3-butanediol, glycolytic fluxes could be successfully modulated to maximize yield and productivity, while minimizing by-product formation in *Escherichia coli*.

**Conclusions:**

These results support the application of this simple method to explore the maximum yield and productivity when designing optimal cell factories for value-added products in the fields of metabolic engineering and synthetic biology.

**Electronic supplementary material:**

The online version of this article (doi:10.1186/s13068-017-0847-4) contains supplementary material, which is available to authorized users.

## Background

Optimal microbial cell factories are essential to develop economically feasible production processes for various value-added chemicals from renewable biomass at an industrial scale [1]. Therefore, the design of cell factories in the fields of metabolic engineering and synthetic biology aims to maximize cellular performance in terms of yield and productivity. This optimization is particularly important for high-volume (and low-value) bulk chemicals and biofuels [2], e.g., *n*-butanol (an alternative to gasoline) [3], butyrate (chemical feedstock for plastics) [4], and 2,3-butanediol (for rubbers) [5].

**Fig. 1:**
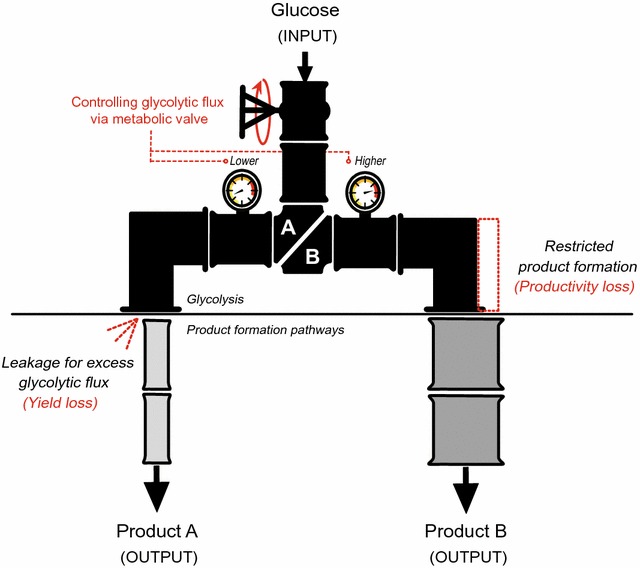
Schematic diagram describing the discrepancy in efficiency between glycolysis and the product-forming pathway and the concept of a metabolic valve. In the general case, the native Flux_Glycolysis_ exceeds the engineered Capacity_Product-forming pathway A_ (width of pipelines), resulting in the leakage of excess carbon as a by-product. In the opposite case, the Capacity_Product-forming pathway B_ is enough to cover glycolytic flux; therefore, overall production rates are determined by Capacity_Glycolysis_. Using a metabolic valve, the glycolytic flux could be tuned down for product A (denoted gage as ‘lower’) or amplified for product B (denoted gage as ‘higher’)

In general, cell factories can be simplified into two parts: a carbon utilization pathway, such as glycolysis, and a product formation pathway (Fig. 1). Traditionally, research in this field has focused on the product-forming pathways of interest. Yield can be enhanced by rerouting the carbon flux toward the target product by eliminating endogenous side reactions, and productivity can be improved by increasing the catalytic activity of kinetic bottlenecks in the product formation pathway [6–8]. However, we additionally speculated that the kinetic imbalance between glycolysis and product formation pathways should be considered in the design principle for optimal cell factories to maximize yield and productivity. When the maximum catalytic activity of the engineered pathway, i.e., the capacity of the product formation pathway, is lower than the glycolytic activity, additional carbon inputs can be wasted as by-products and the yield is consequently reduced (Fig. 1, Product A). In contrast, if the product formation capacity exceeds the glycolytic flux, glycolytic activity can be regarded as the rate-limiting step and increases in activity are necessary to improve productivity (Fig. 1, Product B). Pyruvate, for example, is a critical intermediate between sugar uptake and product formation as a final metabolite of glycolysis in almost all organisms [9]. Excess pyruvate, i.e., quantities that exceed the requirement for product formation, is inevitably secreted from the cell [10, 11], causing a substantial reduction in yield, whereas a lack of pyruvate limits the product formation rate, i.e., reduces productivity (Fig. 1). Taken together, a balance between glycolysis and product formation is required to construct a microbial cell factory with the maximum performance and this can be achieved via the precise control of glycolysis to maintain a balance with the capacity of the product formation pathway [12, 13].

**Fig. 2:**
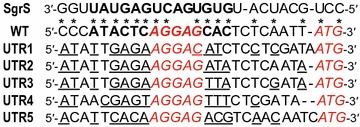
Redesign of the 5′-UTR for *ptsG* based on base-pairs between SgrS and *ptsG*. The* asterisks* indicate the predicted base-pairing region of SgrS required for the translational repression of *ptsG* mRNA [24]. In particular, the minimal base-pairs for SgrS action for effective translational inhibition are shown in *bold* [26, 48]. The *italic letters* represent the Shine–Dalgarno (SD) sequence and the initiation codon for *ptsG*. The changed nucleotides with respect to the wild-type sequence of the *ptsG* UTR are* underlined*

However, methods to control the glycolytic flux are not well-studied owing to the robustness of native glycolytic activity, which is mediated by complex regulatory systems at many levels, including transcription, translation, and the allosteric control of enzymes [14]. Therefore, we focused on the carbon uptake system for the artificial control of carbon influx and simultaneously attempted to detour innate cellular regulatory mechanisms. There are several routes to start glycolysis in bacteria. For example, the phosphoenolpyruvate (PEP)-dependent sugar phosphotransferase system (PTS) that predominantly participates in both the transportation and phosphorylation of glucose. Alternatively, glucose can be internalized by a galactose transporter (GalP or MglABC) and subsequently phosphorylated by hexokinase to enter glycolysis. As such alternative pathways enable to decouple glucose transportation and PEP-dependent phosphorylation, and therefore the pathways were previously exploited to increase precursor availability, such as PEP and free glucose, for the production of aromatic amino acids [15, 16] and gluconic acid [17, 18], respectively. Strikingly, however, the PTS is the most efficient system in terms of energetic costs and kinetic parameters for glucose transportation among the routes [19]. The group translocation system is composed of non-sugar-specific soluble proteins: the phosphohistidine carrier protein (HPr) and the Enzyme I (EI) component (encoded by *ptsH* and *ptsI*, respectively), the glucose-specific cytoplasmic enzyme EIIA (EIIA^Glc^, encoded by *crr*), and the membrane-bound glucose-specific enzyme IICB (EIICB^Glc^, encoded by dicistronic *ptsG*) [19, 20]. Moreover, PTS is primarily responsible for the control of glucose uptake in response to overflow glycolytic flux (for example, the accumulation of glucose-6-phosphate) via the post-transcriptional repression of *ptsG* as the initial step in glucose import [21, 22]. Previous studies revealed that *Escherichia coli* small RNA SgrS is induced under glucose phosphate stress and it causes the translational repression and the RNaseE-dependent rapid degradation of the *ptsG* mRNA [23] by binding to the 5′-end of mRNA [24, 25]. More recent work characterized a minimal base-pairing region between SgrS and *ptsG* mRNA that 14nt base-pairing region including Shine–Dalgarno (SD) sequence of the target mRNA is sufficient to inhibit *ptsG* translation (Fig. 2) [26].

In this study, we examined the potential use of *ptsG* as a simple method to control the overall glycolytic flux simply by designing a synthetic 5′-untranslated region (UTR). UTR engineering is a suitable approach for controlling expression of target genes as well as for eliminating the unpredictable regulatory elements within the metabolic pathway [27]. Furthermore, we demonstrated the importance of rebalancing glycolytic flux depending on the efficiency of product formation pathways using recombinant *E. coli* strains producing *n*-butanol, butyrate, or 2,3-butanediol as model cell factories. Our approach enables the maximization of both yield and productivity in the construction of microbial cell factories by simply optimizing glycolytic flux; accordingly, it has broad applications for the cost-effective production of various chemicals and fuels.

## Methods

### Reagents, bacterial strains, and plasmids

A list of *E. coli* bacterial strains and plasmids used in this study is presented in Additional file 1: Table S1. Oligonucleotides used in this study were synthesized by Macrogen (Daejeon, Korea) and are listed in Additional file 1: Table S2. The *rpsL*-*neo* template DNA was obtained using the Counter-selection BAC Modification Kit (Gene Bridges, Heidelberg, Germany). Phusion DNA Polymerase and restriction endonuclease were supplied by New England Biolabs (Ipswich, MA, USA), and T4 DNA ligase was purchased from Takara Bio Inc. (Shiga, Japan). Genomic DNA and propagated plasmids were prepared using a GeneAll Exgene™ Cell SV Kit (GeneAll Biotechnology, Seoul, Korea) and an AccuPrep Nano-Plus Plasmid Mini Extraction Kit (Bioneer, Daejeon, Korea), respectively. Restriction enzyme-digested products were purified using a GeneAll Expin™ Gel SV Kit (GeneAll Biotechnology). All cell culture reagents were purchased from BD Biosciences (Sparks, MD, USA), and all other chemicals used in this study were purchased from Sigma (St. Louis, MO, USA), unless otherwise indicated.

Chromosomal modifications, including deletions and substitutions of the 5′-UTR of *ptsG*, were performed using the Red recombination system. Specifically, the knock-out mutant of *ptsG* was constructed using the Red recombination system with pKD46 and pCP20 [28, 29]. To increase the efficiency of homologous recombination, disruption cassettes with different priming sites (pFRT 4) were cloned and amplified using ptsG_del4_F and ptsG_del4_R, as described in our previous studies [3, 4]. In addition, the replacement of the native UTR of *ptsG* was performed using the scar-less recombineering method [30] with Red recombination and the *rpsL*-*neo* counterselection system according to the manufacturer’s instructions. For example, a mutation within the *rpsL* gene that confers a streptomycin-resistant phenotype was introduced using rpsL-A128G-oligo. The resulting JHL163 (rpsL*_A128G_) strain was subjected to the insertion of a *rpsL*-*neo* cassette upstream of the *ptsG* structural gene, exhibiting recessive sensitivity to streptomycin in a merodiploid (JHL110). Finally, oligo recombination using [ptsG_UTR(1 to 5)_oligo] that had distinctly redesigned 5′-UTR sequences based on UTR Designer (http://sbi.postech.ac.kr/utr_designer) [31] resulted in the *ptsG* UTR variants, UTR1, 2, 3, 4, and 5, without gaps (Fig. 2). The other strains were constructed in the same manner.

### Media and growth conditions

Physiological analyses were conducted as follows: wild-type *E. coli* was aerobically cultivated using M9 medium (6.78 g of Na_2_HPO_4_, 3 g of KH_2_PO_4_, 1 g of NH_4_Cl, 0.5 g of NaCl, 2 mL of 1 M MgSO_4_, and 0.1 mL of 1 M CaCl_2_/L) supplemented with 40 g/L glucose [32]. Streptomycin (25 μg/mL) was used to determine the genotype of rpsL*_A128G_. Overnight culture broths in LB medium were inoculated at approximately 1% into M9 culture medium and cultivated until reaching an optical density at 600 nm (OD_600_) of ~0.8. The culture broths were inoculated at a final OD_600_ of 0.05 in 25 mL of M9 medium in a 300-mL flask and incubated at 37 °C with shaking (250 rpm). The production of *n*-butanol was assayed using Terrific Broth (TB; 12 g of tryptone, 24 g of yeast extract, 2.31 g of KH_2_PO_4_, 12.54 g of K_2_HPO_4_, and 4 mL of glycerol per liter) supplemented with 25 g/L glucose. Multiple plasmids were maintained using 25 μg/mL spectinomycin and 15 μg/mL kanamycin (pCDF-BuOH and pCOLA-F5). Rubber-sealed, 60-mL serum bottles were used for anaerobic cultures using an anaerobic chamber (Coy Laboratories, Ann Arbor, MI, USA). Overnight culture broths in LB medium were inoculated into 20 mL of fresh TB medium at a final OD_600_ of 0.05 and incubated anaerobically at 37 °C in a rotary shaker (250 rpm) [3]. The production of butyric acid was assayed using Terrific Broth (TB; 12 g of tryptone, 24 g of yeast extract, 2.31 g of KH_2_PO_4_, 12.54 g of K_2_HPO_4_, excluding glycerol) supplemented with 10 g/L glucose. The plasmid (pBASP) was maintained by including 34 μg/mL chloramphenicol. Rubber-sealed, 60-mL serum bottles were used for anaerobic cultures using an anaerobic chamber (Coy Laboratories). Overnight culture broths in LB medium were inoculated into 20 mL of fresh TB medium at a final OD_600_ of 0.05 and incubated anaerobically at 37 °C in a rotary shaker (250 rpm) [4]. The production of 2,3-butanediol was tested using M9 medium (6.78 g of Na_2_HPO_4_, 3 g of KH_2_PO_4_, 1 g of NH_4_Cl, 0.5 g of NaCl, 2 mL of 1 M MgSO_4_, and 0.1 mL of 1 M CaCl_2_/L) supplemented with 40 g/L glucose and 5 g/L yeast extract. The plasmid (pZSbudABC) was maintained by including 30 μg/mL kanamycin. Overnight culture broths in culture medium were inoculated into 100 mL of modified M9 medium at a final OD_600_ of 0.05 and incubated at 37 °C in a rotary shaker (180 rpm) under micro-aerobic condition. The anhydrotetracycline was added to a final concentration of 100 ng/mL when the OD_600_ reached approximately 0.5 [5]. Theoretical yield was determined on the basis of pathway stoichiometry, e.g., 1 mol of *n*-butanol per 1 mol of glucose.

### Analytical methods

The concentrations of glucose, organic acids, and alcohols were determined using high-performance liquid chromatography (UltiMate 3000 Analytical HPLC System; Dionex, Sunnyvale, CA, USA) with an Aminex HPX-87H Column (Bio-Rad Laboratories, Richmond, CA, USA) using 5 mM H_2_SO_4_ as the mobile phase. The 2,3-butanediol samples were analyzed at a flow rate of 0.5 mL/min at 65 °C and otherwise a flow rate of 0.6 mL/min at 14 °C was used to quantify metabolites. The signal was monitored using a UV–Vis diode array detector (at 210 nm) and a Shodex RI-101 detector (Shodex, Klokkerfaldet, Denmark).

### Glucose uptake rate

The specific glucose uptake rate was determined as growth rate divided by biomass yield during exponential growth as previously described [33]. One OD_600_ unit corresponds to 0.25 g dry cell weight (DCW)/L [34]. Instead, glucose consumption rate, determined as the analytic data from HPLC during the initial exponential phase, was represented for the production systems as the components in the TB medium also contributed to biomass yield.

## Results

### Tuning glycolytic activity through UTR engineering of *ptsG*

**Fig. 3:**
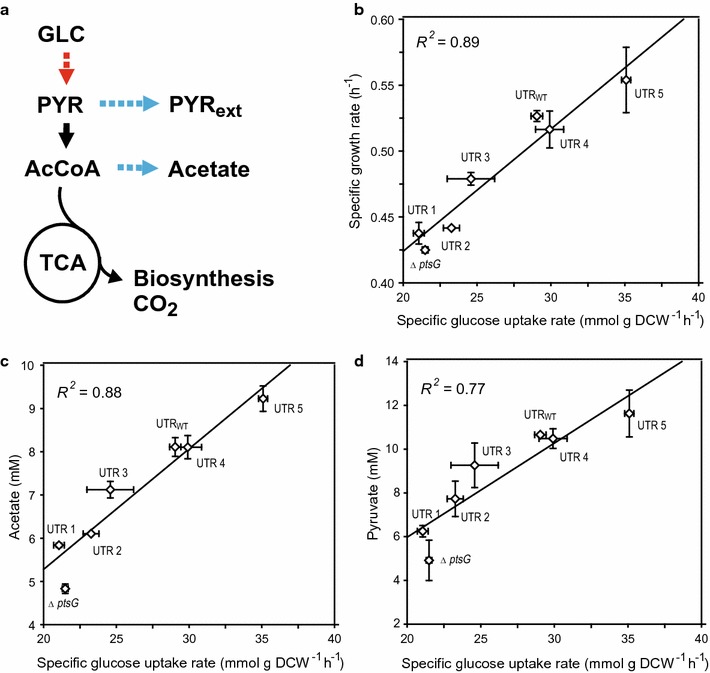
Physiological comparison among *ptsG* variants of wild-type *E. coli* W3110. **a** Schematic metabolic pathway for wild-type *E. coli*. *The dotted red and blue arrows* indicate glycolytic flux control and the corresponding change in flux for two natural metabolites, respectively. **b** Specific growth rate, **c** acetate, and **d** pyruvate are represented as a function of the specific glucose uptake rate. The specific glucose uptake rate was determined as growth rate divided by biomass yield during exponential growth as previously described [33]. One OD_600_ unit corresponds to 0.25 g dry cell weight (DCW)/L. The error bars indicate standard deviations of measurements from two independent cultures

We initially redesigned five 5′-UTR variants to control *ptsG* activity as well as to deregulate translational repression [27] by the bacterial small RNA SgrS (which mediates phosphosugar stress responses) by modifying the minimal base-pairing region essential for SgrS action [26] (Fig. 2). As shown in Fig. 3b, seven strains with UTR variants, including positive (UTR_WT_) and negative (∆*ptsG*) control strains, showed the various specific glucose uptake rates that were highly correlated with specific growth rates (*R* ^2^ = 0.89) in the minimal medium. These results agree with previous continuous culture data indicating that the specific glucose uptake rate increases linearly as a function of the dilution or growth rate [33, 35]. Moreover, differences in the glucose consumption rate were also related to the accumulation of acetate (*R* ^2^ = 0.88) and pyruvate (*R* ^2^ = 0.77) (Fig. 3a, c, d). As the secretion of acetate and pyruvate is generally considered to result from a higher carbon flux than the flux through the TCA cycle, which is required for both biosynthesis and energy production (Fig. 3a) [10, 36], the accumulation of acetate and pyruvate as natural by-products in wild-type *E. coli* collectively represents glycolytic activity. Consequently, our results show that UTR engineering of *ptsG* could successfully modulate overall PTS activity (represented as the glucose uptake rate) and glycolytic flux.

Interestingly, the redesign of the upstream region of *ptsG* enabled a higher specific glucose uptake rate (+20.8%), probably due to the deregulation of SgrS action, and subsequently led to a higher growth rate (+7.3%) and higher accumulation of acetate (+13.9%) and pyruvate (+11.0%) than those of the parental strain (Fig. 3b–d). These results indicate that the glucose transporter (encoded by *ptsG*) can amplify glycolytic flux as a preliminary rate-determining step, even in the presence of complex regulatory mechanisms for other glycolytic enzymes [14].

### Leak-free pathway engineering to improve *n*-butanol cell factory yield

**Fig. 4:**
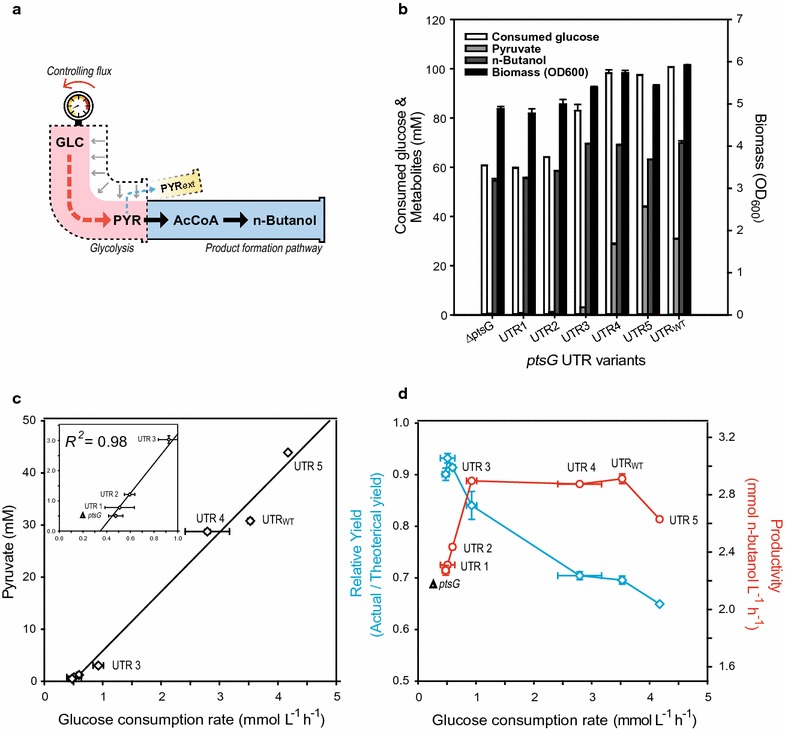
Tuning the glucose uptake rate to improve the yield of *n*-butanol. **a** Schematic metabolic pathway for *n*-butanol fermentation. The* dotted black outer lines* and* red* and* blue arrows* indicate controlled glycolytic flux and the corresponding flux change for pyruvate, respectively. **b** Result of fermentation for *ptsG* UTR variants (Biomass, consumed glucose, *n*-butanol, and pyruvate). **c** Leaking of pyruvate as a function of the glucose consumption rate. **d** Relative yield (*blue*) and productivity (*red*) of *n*-butanol depending on the glucose consumption rate. Relative yield represents the ratio compared to the theoretical maximum yield. From left, the *open circle* represents ∆*ptsG*, UTR1, UTR2, UTR3, UTR4, UTR_WT_, and UTR5. All data were obtained after fermentation for 24 h in TB medium. It should be noted that the components in the TB medium also contributed to biomass yield; therefore, glycolytic flux was represented as the glucose consumption rate during the initial exponential phase, rather than the specific glucose uptake rate. The *error bars* indicate standard deviations of measurements from two independent cultures

The *n*-butanol synthetic pathway was selected as an example in which glycolytic activity was higher than product formation under anaerobic condition. Previously, many studies have attempted to optimize the *n*-butanol production pathway, but substantial levels of pyruvate accumulated as a by-product in the medium, indicating the *n*-butanol synthesis pathways are still inefficient [3, 37, 38]. Therefore, in this case, tuning down of glycolytic flux is an effective way to minimize wasteful pyruvate production, which decreases yield (Fig. 4a).

We used an approach that we termed leak-free pathway engineering to improve the yield of the *n*-butanol cell factory. Seven *ptsG* UTR variants, including the native sequence (JHL 178–183), were engineered using *n*-butanol-producing *E. coli* JHL 59 (Δ*ato*DA Δ*adhE* Δ*ldh*A Δ*paa*FGH Δ*frd*ABCD Δ*pta* P_*ato*B_::BBa_J23100 P_*lpd*_::BBa_J23100 *lpd*(G1060A) P_*aceEF*_::BBa_J23100) as the parental strain [3]. After a 24-h fermentation period, each variant showed different physiological results in terms of the accumulation of biomass, *n*-butanol, and pyruvate as well as glucose consumption (Fig. 4b). Along with the decrease in the glucose consumption rate, 99% of pyruvate secretion was successfully eliminated, from 43.92 mM in UTR5 (JHL179) to 0.50 mM in ∆*ptsG* (JHL184), by glycolytic flux modulation, and there was a strong correlation between pyruvate secretion and the glucose consumption rate (*R* ^2^ = 0.98) (Fig. 4c). The final titer of *n*-butanol decreased from 69.88 mM (UTR_WT_, JHL178) to 54.54 mM (∆*ptsG*, JHL184) (Fig. 4b). In addition, the specific growth rate showed strong correlations between the glucose consumption rate (*R* ^2^ = 0.93) and the specific glucose uptake rate (*R* ^2^ = 0.94), even in rich TB medium (Additional file 1: Figures S1, S2, respectively). These results indicate that controlling the *ptsG* expression level through UTR engineering could successfully modulate the glycolytic flux of the engineered strain, even under anaerobic conditions.

To evaluate cellular performance in *n*-butanol production, yield and productivity were examined (Fig. 4d). Notably, *n*-butanol yield increased as glycolytic flux decreased, which was attributed to a reduction in pyruvate leakage (Fig. 4c), but only slight changes in productivity were observed. This clearly shows that reducing glycolytic flux by modifying the glucose uptake rate had a greater influence on pyruvate secretion than *n*-butanol production. Among the tested variants, the JHL181 strain with the UTR3 variant indicated the optimal glycolytic flux for the best trade-off between yield and productivity as it showed 84% of the theoretical maximum yield by a 20% improvement (0.84 mol butanol/mol glucose) compared to the parental strain, but exhibited negligible changes in productivity (2.90 mM butanol L/h for UTR3 vs. 2.91 mM butanol L/h for UTR_WT_) (Fig. 4d). Under the level of UTR3, however, *n*-butanol productivity decreased as a function of the glucose uptake rate, even though the yield increased to 93% of the theoretical maximum (Please see UTR2 in Fig. 4d). This indicates that glycolytic flux with UTR3 corresponds to the capacity of the engineered *n*-butanol synthesis pathway and glycolytic fluxes below this level can be regarded as the rate-limiting step for the production of *n*-butanol (Fig. 4d). The JHL179 strain with the UTR5 variant, whose rate of glucose uptake was higher (+18.34%) than that of the parental strain, showed substantial reductions in yield as well as productivity owing to a significant decrease in pH resulting from acidic pyruvate accumulation (+42.65% compared to UTR_WT_; Fig. 4c), which negatively affected glucose consumption (Fig. 4b). Taken together, our results demonstrate that yield can be maximized while maintaining maximum productivity simply by optimizing the glycolytic flux according to the capacity of the product formation pathways via fine-control of *ptsG*.

### Improvement in productivity by enhanced glycolytic activity through UTR engineering of *ptsG*

In general, product yield can be maximized via the deletion of pathways for unnecessary by-product formation, but increasing productivity beyond this maximized yield is challenging [7]. Nevertheless, further increases in productivity, while maintaining the maximum yield can be expected by enhancing the glycolytic flux if the capacity of the product formation pathway is higher than the natural glycolytic activity.

**Fig. 5:**
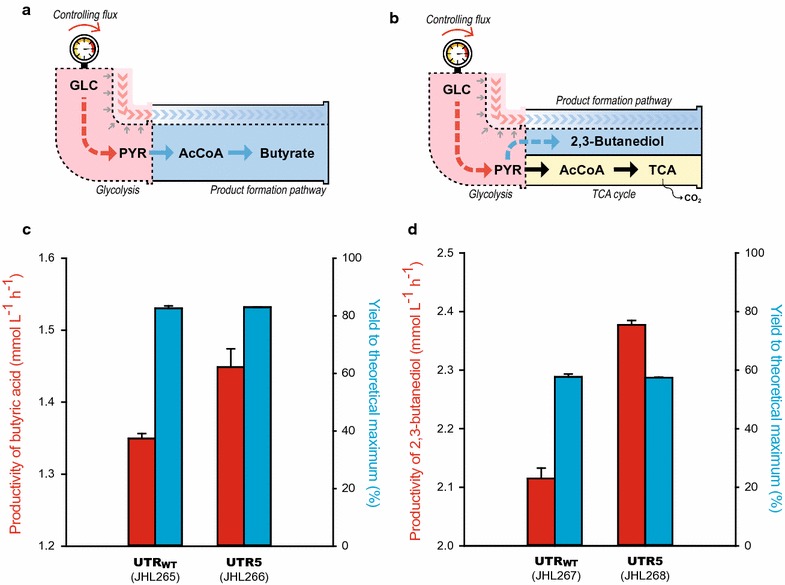
Comparative analysis of UTR5 to improve the productivity of butyrate and 2,3-butanediol. **a** Schematic metabolic pathway for the production of **a** butyrate and **b** 2,3-butanediol. The flux for the TCA cycle was included in the 2,3-butanediol system due to the presence of oxygen. *The dotted red and blue arrows* indicate controlled glycolytic flux and additional carbon flux from glycolysis to the product-forming pathway, respectively (*dotted black outer lines*). **c** The productivity (*red*) and yield (*blue*) of butyrate were compared after 24 h of fermentation in TB medium. **d** The productivity (*red*) and yield (*blue*) of 2,3-butanediol were calculated after 39 h of cultivation in modified M9 medium. The *error bars* indicate standard deviations of measurements from two independent cultures

To verify this, previously engineered *E. coli* strains for the production of butyrate [4] and 2,3-butanediol [5] were exploited as model systems; their product yields were close to the theoretical maximum due to the elimination of native by-product formation pathways, such as lactate and ethanol. Since butyrate is a fermentative product, energy for biosynthesis is mostly generated by the butyrate production pathway under anaerobic conditions, and the engineered strain JHL265 showed 83.4% of the theoretical maximum yield (Fig. 5a) [4]. However, the biological conversion rate of 2,3-butanediol from pyruvate could be maximized in the presence of oxygen and therefore a portion of the carbon source should be consumed to generate energy by conversion to carbon dioxide (Fig. 5b) [39].

To enhance glycolytic activity, *ptsG* expression was activated using UTR5 (resulting JHL266). As expected, the amplified glucose consumption rate translated to 7% higher productivity for butyrate (1.45 mmol butyrate L/h) than the parental strain, while the yield was maintained at approximately 83% of theoretical maximum (Fig. 5c). In the case of 2,3-butanediol production, the productivity of the strain with higher glycolytic activity (UTR5), resulting JHL268, could be improved by 12.45% compared to the parental strain JHL267 (2.38 mmol 2,3-butanediol L/h), while maintaining the parental maximum yield (approximately 60% of the theoretical maximum), as shown in Fig. 5d. Our results clearly show that the productivity of biological processes could be improved by amplifying glycolysis *per se* through UTR engineering of *ptsG*.

## Discussion

Although the entire pathway from sugar uptake to product formation has to be well-balanced for optimal yield and productivity, research in metabolic engineering has focused on production pathways. Furthermore, controlling glycolytic flux remains a daunting task owing to incomplete knowledge of the mechanisms that regulate glycolysis [14]. While many process control techniques, such as carbon limited fed-batch cultivation, are the standard approaches to control overflow metabolism [40], our approach has the advantage of increasing robustness of biological production by optimizing glycolytic flux at the genetic level.

In this study, we demonstrated the physiological relevance of *ptsG* to overall glycolytic activity as the simple method for the control of metabolic input. As small RNA SgrS inherently represses the translation of *ptsG* mRNA by sequestering its ribosome binding site and RNaseE-dependent cleavage through a short base-pairing interaction [21, 22], the glucose transporter encoded by *ptsG* was modulated using synthetic 5′-UTRs for the fine-control of translation efficiency in addition to the deregulation of SgrS. Although the molecular study for the UTR engineering-mediated mitigation of SgrS regulation should be further investigated, our physiological results successfully demonstrate the ability to control the glycolytic flux through *ptsG* as shown in Fig. 3. Moreover, the redesign of native UTR for allowed an increase in glycolytic flux by 20.8% compared to the wild type, even though none of the overexpressed glycolytic enzymes increased glycolytic activity in previous studies [41–43]. Since native glycolytic activity is often not sufficient for non-native product formation pathways and therefore increased glycolytic activity is necessary to maximize the rate of product formation for industrial applications, the observation that *ptsG* might be a preliminary rate-determining step in glycolysis is also intriguing.

Using these findings, the optimal glycolytic flux was explored with respect to the capacity of the *n*-butanol, butyrate, and 2,3-butanediol synthesis pathways to improve cellular performance. Interestingly, the yield of *n*-butanol increased to 93% of the theoretical maximum due to a reduction in pyruvate secretion in accordance with tuning down of the glycolytic flux. Conversely, enhanced productivity was observed for the production of butyrate and 2,3-butanediol by activating the expression level of *ptsG* (via UTR5). Collectively, these results clearly indicate that optimization of the glycolytic flux enables additional improvements in both yield and productivity of cell factories, beyond optimization of the product formation pathway.

**Fig. 6:**
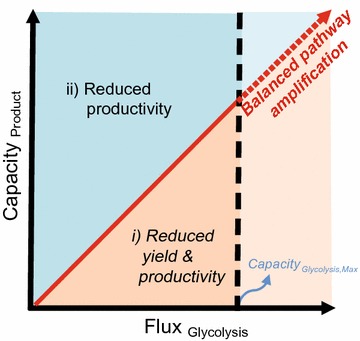
Plausible scenarios regarding the efficiencies of the two pathways: (*i*) Reduced yield and productivity (Flux_Glycolysis_ > Capacity_Product-forming pathway_). (*ii*) Maximized yield, but reduced productivity due to glycolytic flux, which itself acts as a rate-limiting step (Flux_Glycolysis_ < Capacity_Product-forming pathway_). Further improvement in productivity is restricted when the product-forming pathway exceeds the upper biological constraint (Flux_Glycolysis, Max_ < Capacity_Product-forming pathway_). The* faded region* has never been explored. The* red arrow* indicates the optimized conditions for both glycolysis and the product-forming pathway (Balanced pathway, see “Discussion”)

The concept of optimizing glycolytic flux is also important to the microbial production of various chemicals and fuels from cost-effective feedstock, such as glycerol [44] and galactose [45] and our strategy can be applied to explore optimal glycolytic flux depending on the capacity of product formation pathway via fine-control of glycerol transporter (encoded by glpF) [46] and galactose transporter (encoded by galP) [47], respectively. Ultimately, as summarized in Fig. 6, balanced pathway amplification of both glycolytic flux and product-forming pathways are highly desirable for the design of economically feasible microbial cell factories in the bio-based chemical industry.

## Conclusions

In this study, we examined the metabolic imbalance between glycolysis and product formation pathways using recombinant *Escherichia coli* strains producing *n*-butanol, butyrate, or 2,3-butanediol as model cell factories. Initially, the glucose uptake rate of wild-type *E. coli* was fine-tuned using synthetic UTRs of *ptsG* to modulate the overall glycolytic fluxes, which were assessed by physiological parameters, i.e., specific growth rate and the accumulation of acetate and pyruvate as natural by-products. Moving forward, glycolytic flux was rebalanced via the control of *ptsG* depending on the efficiency of product formation pathways with lower (*n*-butanol) and higher (butyrate and 2,3-butanediol) product formation capacities compared to the wild-type glycolytic flux. For the production of *n*-butanol, glycolytic flux successfully tuned down to minimize by-product formation, while maintaining productivity, which we termed leak-free pathway engineering. Conversely, butyrate and 2,3-butanediol production rates were increased using a UTR variant of *ptsG* with higher glycolytic flux than that of the wild type. These results demonstrate the simple method to control glycolytic flux for the design of optimal cell factories in the fields of metabolic engineering and synthetic biology.

## Additional file

**Additional file 1.** Additional figures, tables and reference.

## Abbreviations

DCW: dry cell weight
OD: optical density
PEP: phosphoenolpyruvate
PTS: phosphotransferase system
TCA: tricarboxylic acid
UTR: untranslated region
