# Supplementary material for: A simple method to control glycolytic flux for the design of an optimal cell factory
Source: Biotechnol Biofuels. 2017 Jun 21;10:160. doi: 10.1186/s13068-017-0847-4 (PMC5480111; doi:10.1186/s13068-017-0847-4)
Supplement: Supplementary file 1 — Additional file 1. Additional figures, tables and reference. [file 13068_2017_847_MOESM1_ESM.doc]

**Additional Information for:**

Jae Hyung Lim, Gyoo Yeol Jung^*^

* To whom correspondence should be addressed.

E-mail: gyjung@postech.ac.kr (G. Y. J.)

**Contents:**

**Additional Figures and Legends**

**Additional Tables**

**Additional References**

**Additional Figures and Legends**

**Figure S1. Specific growth rate of *n*-butanol-producing *E. coli*.**

Specific growth rate of *n*-butanol-producing *E. coli* under rich TB medium depicted in order of glucose consumption rate. The error bars indicate standard deviations of measurements from two independent cultures.

**Figure S2. Specific glucose uptake rate of n-butanol-producing *E. coli*.**

The data were calculated from glucose consumption rate and biomass under rich TB medium.

It should be noted that the components in the TB medium also contributed to biomass yield. The error bars indicate standard deviations of measurements from two independent cultures.

**Additional Tables**

**Table S1.** Bacterial strains and plasmids used in this study.

| **Name** | **Relevant characteristics** | **Source** |
| --- | --- | --- |
| **Strain** |  |  |
| Mach1-T1^R^ | F^-^ φ80(*lac*Z)ΔM15 Δ*lac*X74 *hsd*R(r_K_^-^m_K_^+^) Δ*rec*A1398 *end*A1 *ton*A | Invitrogen |
| W3110 | F^-^ λ^-^ *rph-1IN*(*rrnD, rrnE*)*1* | ATCC 27325 |
| JHL163 | W3110 *rpsL**_A128G_ (Str^R^) | This study |
| JHL110 | JHL163 *ptsG:*:*rpsL*-*neo* (Str^S^, Kan^R^) | This study |
| JHL164 | JHL163 *ptsG* _UTR5_ | This study |
| JHL165 | JHL163 *ptsG* _UTR4_ | This study |
| JHL166 | JHL163 *ptsG* _UTR3_ | This study |
| JHL167 | JHL163 *ptsG* _UTR2_ | This study |
| JHL168 | JHL163 *ptsG* _UTR1_ | This study |
| JHL169 | JHL163 Δ*ptsG* | This study |
| JHL59 | W3110 Δ*ato*DA Δ*adhE* Δ*ldh*A Δ*paa*FGH Δ*frd*ABCD Δ*pta* P*_ato_*_B_::BBa_J23100 P*_lpd_*::BBa_J23100 *lpd*(G1060A) P*_aceEF_*::BBa_J23100 |  |
| JHL170 | JHL59 *rpsL**_A128G_ (Str^R^) | This study |
| JHL178 | JHL170 / pCDF-BuOH, pCOLA-F5 | This study |
| JHL179 | JHL170 *ptsG* _UTR5_ / pCDF-BuOH, pCOLA-F5 | This study |
| JHL180 | JHL170 *ptsG* _UTR4_ / pCDF-BuOH, pCOLA-F5 | This study |
| JHL181 | JHL170 *ptsG* _UTR3_ / pCDF-BuOH, pCOLA-F5 | This study |
| JHL182 | JHL170 *ptsG* _UTR2_ / pCDF-BuOH, pCOLA-F5 | This study |
| JHL183 | JHL170 *ptsG* _UTR1_ / pCDF-BuOH, pCOLA-F5 | This study |
| JHL184 | JHL170 Δ*ptsG* / pCDF-BuOH, pCOLA-F5 | This study |
| JHL265 | JHL170 / pBASP | This study |
| JHL266 | JHL170 *ptsG* _UTR5_/ pBASP | This study |
| JHL267 | JHL170/ pZSbudABC | This study |
| JHL268 | JHL170 *ptsG* _UTR5_/ pZSbudABC | This study |
|  |  |  |
|  |  |  |
|  |  |  |
|  |  |  |
|  |  |  |
|  |  |  |
|  |  |  |
|  |  |  |
|  |  |  |
|  |  |  |
| **Plasmid** |  |  |
| pKD4 | Template plasmid for FRT-flanked kanamycin resistance gene; Amp^R^, Km^R^ |  |
| pKD46 | Red recombinase expression vector; Amp^R^ |  |
| pCP20 | FLP expression vector; Amp^R^ |  |
| pCR2.1-TOPO | Cloning vector, Amp^R^, Km^R^ | Invitrogen |
| pMD20-T | Cloning vector, Amp^R^ | Takara |
| pGEM T-Easy | Cloning vector, Amp^R^ | Promega |
| pFRT4 | From pGEM T-Easy, FRT*-Kan^R^-*FRT(4) | This study |
| pCDF-BuOH | cloDF13 ori, Sm^R^, P_J23100_::*crt*- P_J23100_::*hbd*- P_J23100_::*ter*-P_J23100_::*adhE2* |  |
| pCOLA-F5 | ColA ori, Km^R^, P_J23100_::F5_UTR_- *fdh1*_SC_ |  |
| pBASP | p15A ori, Cm^R^, P_J23100_::*crt*- P_J23100_::*hbd*- P_J23100_::*ter*-P_J23100_::*tesB* |  |
| pZSbudABC | pSC101 ori, Km^R^, *Enterobacter aerogenes bud* ABC operon under the control of P_LtetO-1_, |  |
|  |  |  |
|  |  |  |

**Table S2.** Primers used in this study.

| **Name^a^** | **Sequence (5′-3′)^b,c^** |
| --- | --- |
| FRT4_F | ctagtgctggagcgaactgcgaagttcctatactttctagagaataggaacttcggaataggaacttcaagatcccctcacgctgccgc |
| FRT4_R | ggagtactcgcggttgactgagttcctattccgaagttcctattctctagaaagtataggaacttcagagcgcttttgaagctggggtg |
| ptsG_del4_F | cgttgtatcgcatgttatggcagaagcaggcggttccgtctttgcaaacactagtgctggagcgaactgc |
| ptsG_del4_R | aacgctgacgcgcagacgggtaatacatgcgtcgaggttagtaatgttttggagtactcgcggttgactg |
| rpsL-A128G-oligo | cgttagtcagacgaacacggcatactttacgcagcgcggagttcggttttctaggagtggtagtatatacacgagtacatacgccacgtttttgcgggcat |
| ptsG_rpsLneo_F | acacggcgaggctctccccccttgccacgcgtgagaacgtaaaaaaagcaggcctggtgatgatggcggg |
| ptsG_rpsLneo_R | atcagcgatttaccgaccttttgcaggttagcaaatgcattcttaaacattcagaagaactcgtcaagaaggcgatagaag |
| ptsG_UTR1_oligo | acacggcgaggctctccccccttgccacgcgtgagaacgtaaaaaaagca***ATATTGAGAAGGACATCTCCTCGATA***atgtttaagaatgcatttgctaacctgcaaaaggtcggtaaatcgctgat |
| ptsG_UTR2_oligo | acacggcgaggctctccccccttgccacgcgtgagaacgtaaaaaaagca***ATATTGAGAAGGAGATATCTCAATA***atgtttaagaatgcatttgctaacctgcaaaaggtcggtaaatcgctgat |
| ptsG_UTR3_oligo | acacggcgaggctctccccccttgccacgcgtgagaacgtaaaaaaagca***ATATTGAGAAGGAGTTATCTCGATA***atgtttaagaatgcatttgctaacctgcaaaaggtcggtaaatcgctgat |
| ptsG_UTR4_oligo | acacggcgaggctctccccccttgccacgcgtgagaacgtaaaaaaagca***ATAACGAGTAGGAGTTTCTCGATA***atgtttaagaatgcatttgctaacctgcaaaaggtcggtaaatcgctgat |
| ptsG_UTR5_oligo | acacggcgaggctctccccccttgccacgcgtgagaacgtaaaaaaagca***ACATTCACAAGGAGACGTCAACAATC***atgtttaagaatgcatttgctaacctgcaaaaggtcggtaaatcgctgat |
| C-ptsG_UTR-F | catatgttttgtcaaaatgtgcaacttctccaatgat |
| C-ptsG_UTR-R | ttttaaccatgatgccataggcaacaactgc |
| C-ptsG_del-F | cggtaaatcgctgatgctgccggta |
| C-ptsG_del-R | gtggttacggatgtactcatccatctcg |

^a^Primer names beginning with “C” indicate primers used to check for homologous recombination. ^b^Underlined letters indicate different priming sequences used to increase the efficiency of homologous recombination. ^c^Italicized, bold letters indicate redesigned target gene 5′-UTR sequences.

**Additional References**

1. Lim JH, Seo SW, Kim SY, Jung GY. Model-driven rebalancing of the intracellular redox state for optimization of a heterologous n-butanol pathway in *Escherichia coli*. Metab Eng. 2013; 20:49-55.

2. Datsenko KA, Wanner BL. One-step inactivation of chromosomal genes in *Escherichia coli* K-12 using PCR products. Proc Natl Acad Sci USA. 2000; 97(12):6640-5.

3. Lim JH, Seo SW, Kim SY, Jung GY. Refactoring redox cofactor regeneration for high-yield biocatalysis of glucose to butyric acid in *Escherichia coli*. Bioresour Technol. 2013; 135:568-73.

4. Mazumdar S, Lee J, Oh MK. Microbial production of 2,3-butanediol from seaweed hydrolysate using metabolically engineered *Escherichia coli*. Bioresour Technol. 2013; 136:329-36.
